# Conditional Knockdown of Osteopontin Inhibits Breast Cancer Skeletal Metastasis

**DOI:** 10.3390/ijms20194918

**Published:** 2019-10-04

**Authors:** Marineta Kovacheva, Michael Zepp, Muriel Schraad, Stefan Berger, Martin R. Berger

**Affiliations:** 1 German Cancer Research Center (DKFZ), Toxicology and Chemotherapy Unit, 69120 Heidelberg, Germany; m.kovacheva@dkfz.de (M.K.); m.zepp@dkftz.de (M.Z.); muriel.schraad@googlemail.com (M.S.); 2 Central Institute of Mental Health, Department of Molecular Biology, 68159 Mannheim, Germany; zebiraf@gmail.com

**Keywords:** conditional osteopontin knockdown, tet-off system, MDA-MB-231 human breast cancer cells, skeletal metastasis, erufosine

## Abstract

High osteopontin (OPN) expression is linked to breast cancer bone metastasis. In this study we modulated osteopontin levels conditionally and investigated any related antineoplastic effects. Therefore, we established cell clones from human breast cancer MDA-MB-231 cells, in which the expression of OPN is regulated by the Tet-Off tet-off system. These cells, which conditionally express a specific miRNA targeting OPN, were used for in vitro studies as well as for a bone metastasis model in nude rats. Changes in whole-genome expression elicited by conditional OPN knockdown and vesicle formation were also analyzed. The alkylphosphocholine erufosine was used for combination therapy. Conditional OPN knockdown caused mild anti-proliferative, but more intensive anti-migratory and anti clonogenic effects, as well as partial and complete remissions of soft tissue and osteolytic lesions. These effects were associated with specific gene and protein expression modulations following miRNA-mediated OPN knockdown. Furthermore, high levels of OPN were detected in vesicles derived from rats harboring breast cancer skeletal metastases. Finally, the combination of OPN inhibition and erufosine treatment caused an additive reduction of OPN levels in the investigated breast cancer cells. Thus, knockdown of OPN alone or in combination with erufosine is a promising strategy in breast cancer skeletal metastasis treatment.

## 1. Introduction

Bone metastasis is pathological process which occurs in about 70% of breast cancer patients with progression of their disease, and causes disturbing consequences including bone pain, pathologic fractures, hypercalcemia, alteration of hematopoiesis, and spinal cord compression. To date, there is no curative treatment available for the metastatic stage of this disease [1]. A deeper knowledge on the mechanisms of bone metastasis and finding of specific targets involved in this process would improve the development of efficient therapies. In this regard, it is important to define the driver genes which are coding these targets, as they presumably have a major impact on breast cancer skeletal metastasis development.

The PI3K pathway is known to be mutated in breast cancer [2,3,4]. Recently, alkylphosphocholines like miltefosine and erufosine have been found active in treating secondary and primary breast cancers [5,6]. They are membrane-seeking agents and interfere with signal transduction pathways. Part of their activity concentrates on the PI3K/Akt/mTOR pathway. Recently, new aspects regarding their effect on cell cycle and on endoplasmic reticulum (ER) stress have been published [7,8]. Another target which has emerged recently is osteopontin. High expression of osteopontin (OPN) in the tumor tissue and plasma of breast cancer patients has been associated with ominous prognosis and reduced survival in these patients [9,10]. OPN is a secreted extracellular glycophosphoprotein with a molecular size of 34 kDa, but after post-translational modifications (including glycosylation, phosphorylation, sulfation, cross-linking, and proteolytic processes) its apparent molecular size varies between 44 and 75 kDa in mammalian cells [10,11]. OPN is also known as secreted phosphoprotein 1 (SPP1), which is assigned to the small integrin-binding ligand *n*-linked glycoprotein (SIBLING) family. These proteins share some structural similarities, such as the integrin-binding tripeptide Arg–Gly–Asp (RGD), as well as several post-translational phosphorylation and *N*-glycosylation sites [11]. OPN is expressed in osteoclasts, osteoblasts, vascular and skeletal smooth muscle cells, endothelial cells, lymphocytes, neural cells, and certain carcinoma cells [10,11]. Because it is secreted by producing cells, OPN is present in the extracellular matrix of mineralized tissues, as well as in body fluids like blood, milk, bile, seminal fluid, and saliva [11]. OPN plays a role in bone remodeling and mediates the interaction between osteoclasts and mineral matrix in the skeleton [12]. It binds to integrins and CD44 receptors, takes part in cell–matrix interactions, including cell signaling, and is related to tumor growth and development [11] as well as cancer skeletal metastasis [9]. Notably, high OPN expression is exhibited in breast cancer bone metastasis [9]. In addition, it has been suggested that tumor-derived OPN lacks some domains. Therefore, it is structurally different to other types of OPN, and blocking its production could be used to reduce tumor progression [12].

Thus, we hypothesized that OPN could serve as a promising target for therapy of breast cancer skeletal metastasis [13]. In order to elucidate the effect of OPN, we intended to use its prolonged knockdown for describing the precise role of OPN in mediating breast cancer progression and metastasis. To that purpose, a model was established which realized a conditional knockdown by using doxycycline-dependent miRNA expression. In this approach, a tet-regulated expression gene cassette, containing an artificial miRNA targeting OPN mRNA, was integrated into a predetermined genomic locus of MDA-MB-231 breast cancer cells by Flp-recombinase-mediated cassette exchange (RMCE). In the absence of doxycycline, the expression of the specific and efficient miRNA was activated in these cell clones, which allowed the convenient monitoring of effects related to OPN inhibition: the observation started at a defined point in time and lasted for a specified period. The established cell clones were used to investigate the effects of OPN knockdown on cellular properties (in vitro) and on breast cancer skeletal metastasis (in vivo), as well as on gene modulation (in vitro). Furthermore, in order to reveal one of the probable mechanisms for metastasis and the role of OPN, its presence was analyzed in exosomes, which were isolated from the plasma of rats with breast cancer skeletal metastasis. Exosomes are small membrane vesicles (30–100 nm) containing proteins, lipids, RNA, and DNA that can be horizontally transferred to recipient cells. Tumor-derived exosomes which are taken up by cells from specific organs could prepare the pre-metastatic niche [14]. Finally, the combination effect of conditional OPN knockdown and erufosine treatment was investigated in breast cancer cells.

## 2. Results

### 2.1. Generation and Characterization of Cell Clones

**Figure 1:**
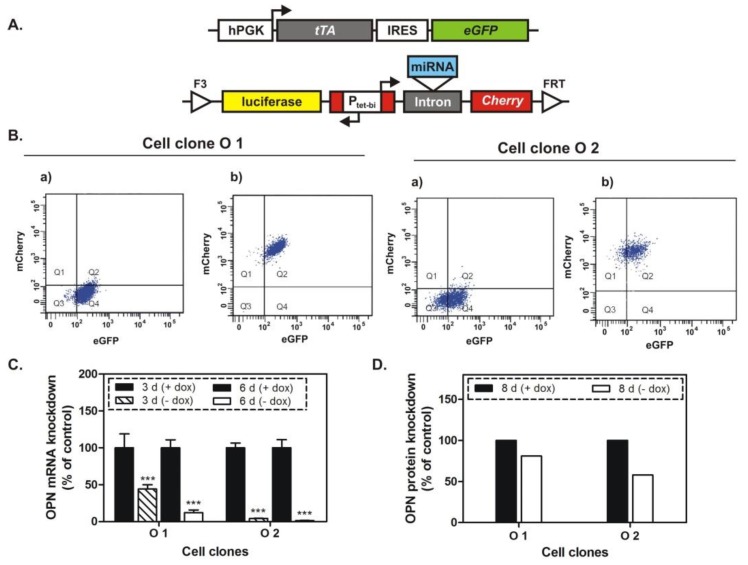
The expression of osteopontin is regulated by a specific miRNA, which is expressed in the absence of doxycycline (dox). (**A**) Two gene cassettes were integrated into the genome of MDA-MB-231 breast cancer cells and thus generated the double transgenic cell clones O1 and O2; Abbreviations used: hPGK—human phosphoglycerate kinase promoter; tTA—tetracycline-controlled transactivator; IRES—internal ribosome entry site; eGFP—enhanced green fluorescent protein; luciferase—firefly luciferase; Ptet-bi—bidirectional tet-regulated promoter; Cherry—red fluorescent protein mCherry; FRT and F3—wild type and mutant; Flp—recombinase target sites. (**B**) Flow cytometry analysis of the transgenic cell clones O1 and O2; (**a**) Fluorescence from cell clones cultivated in medium with doxycycline; (**b**) mCherry expression was stimulated upon removal of doxycycline from the cultivation medium of the cell clones and indicates the concomitant expression of the specific miRNA; (**C**) qPCR analysis of osteopontin (OPN) in O1 and O2 cell clones, cultivated for 3 and 6 days in media with or without doxycycline. OPN mRNA levels were significantly decreased, especially after 6 days of conditional miRNA-mediated OPN knockdown. (**D**) Western blot analysis of OPN in O1 and O2 cell clones, cultivated for 8 days in media with or without doxycycline.

The role of OPN in breast cancer skeletal metastasis was investigated by using two double transgenic MDA-MB-231 cell clones (O1 and O2). Their generation was similar to the previously described procedure [15]. Briefly, two gene cassettes were integrated into the genome of these cells (Figure 1A). One of them regulates the constitutive expression of the tetracycline-controlled transactivator (tTA), which activates the bidirectional promoter Ptet-bi present in the second gene cassette. In the absence of doxycycline, tTA activates this promoter, which stimulates the simultaneous expression of firefly luciferase, red fluorescent protein mCherry, and a highly efficient and specific miRNA targeting OPN mRNA. In the presence of doxycycline, a conformational change of the tTA protein is caused by its binding to the tetracycline derivative, which prevents the interaction between tTA and the tet-regulated promoter. Thus, in this condition, the expression of the respective Ptet-bi-regulated genes will be switched off.

**Figure 2:**
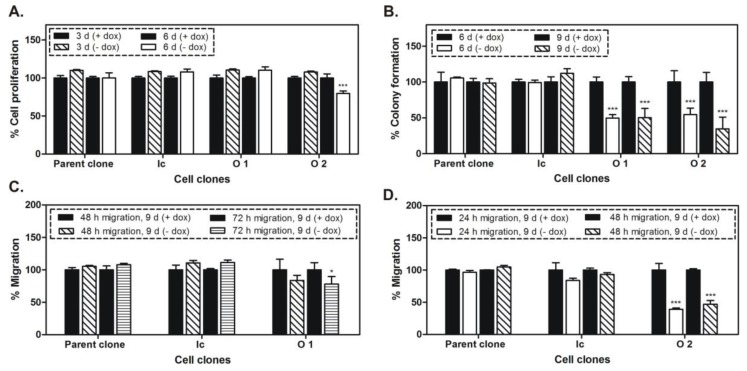
Cellular properties upon conditional OPN knockdown. (**A**) Cell proliferation assay (MTT assay); (**B**) Colony formation assay; (**C**,**D**) Migration assay. The parent cell clone and a cell clone (Ic) containing the gene cassette without the specific miRNA targeting OPN were used as an additional controls to the + dox controls of the cell clones O1 and O2. The proliferation was significantly decreased in cells of the O2 clone only, but the colony formation and migration abilities were inhibited in both cell clones, although more pronounced in O2 cells. * *p* < 0.05; *** *p* < 0.001.

The doxycycline-dependent regulation of the reporter gene mCherry was investigated in the cell clones O1 and O2 by flow cytometry analysis. As shown in Figure 1B, the expression of this tet-regulated gene was increased two times in the absence of doxycycline. Furthermore, the OPN knockdown was analyzed at mRNA and protein levels by real-time PCR (qPCR) and Western blot, respectively, following 3, 6, or 8 days of cultivation in media with or without doxycycline. The exposure of the cell clones to miRNA against OPN resulted in gene silencing characterized by 57–96% and 88–98% inhibition of OPN-mRNA expression after 3 and 6 days, respectively (Figure 1C). The OPN protein level, monitored after 8 days of treatment with the respective miRNA, was decreased by 20% (O1 cell clone) and 43% (O2 cell clone) (Figure 1D). Next, cellular properties related to cancer growth, including proliferation, colony formation, and migration were investigated in the cell clones following conditional OPN knockdown, as well as in two control MDA-MB-231 cell clones (parent and Ic cell clones). These control clones harbor similar gene cassettes, but without the specific miRNA targeting OPN. The inhibition of OPN caused a 20% reduced proliferation in O2 cells only (Figure 2A). Following 6 and 9 days of OPN knockdown, a colony formation assay was performed over a period of 7 days in media with or without doxycycline. As shown in Figure 2B, the miRNA-mediated OPN inhibition resulted in significantly decreased colony formation in O1 and O2 cells of 50% and 46% (6 days) and 50% and 65% (9 days), respectively. Additionally, the cellular migration was reduced by 22% (O1 cell clone) and 53% (O2 cell clone) after 9 days of OPN knockdown (Figure 2C,D). In parallel, no significant changes were detected regarding the proliferation, colony formation, and migration of the control cell clones (parent and Ic clones).

### 2.2. Gene Modulation upon OPN Knockdown

Gene expression profiling was performed in O2 cells after 3 or 6 days of miRNA expression, which was used for targeting OPN. The microarray analysis showed that there were 16 protein-coding genes, which were modulated more than 1.5-fold after three days of conditional OPN knockdown. From these, ten were up-regulated and six were down-regulated. After six days of OPN knockdown, there were 30 protein-coding genes modulated more than 1.5-fold. From these, 50% were up-regulated and 50% were down-regulated. The expression fold change ranged from −1.78 to 1.78 (3 days) and from −2.3 to 1.78 (6 days). In contrast with these numbers, the SPP1 gene was down-regulated 10-fold after both time periods.

**Figure 3:**
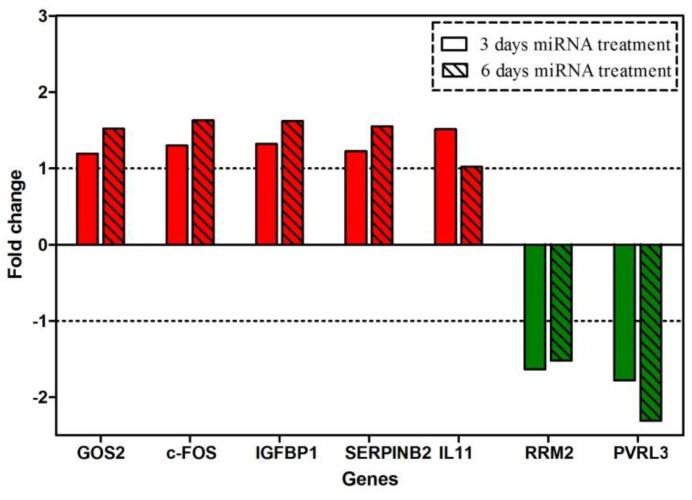
Microarray expression profiling data. Fold change of selected genes’ expression in response to targeting OPN for 3 or 6 days by miRNA treatment. Red bars show the fold changes of genes which were persistently up-regulated (*GOS2*, *c-FOS*, *IGFBP1*, *SERPINB2*) or of a gene whose expression decreased back to the control level (*IL11*); Green bars indicate the fold changes of genes which were persistently down-regulated (*RRM2*, *PVRL3*). Dashed lines indicate the no-effect level regarding gene expression modulation.

An overview of changes in gene expression is given in Table 1. The genes are categorized in three groups according to their modulation (regarding the onset and duration of modulation). The majority of these genes were modulated only at 6 days after OPN knockdown, and they were termed the group of genes with “long-term changes”. They include *IGFBP1*, *SERPINB2*, *c-FOS*, *GOS2*, which were up-regulated more than 1.5-fold. Another group included genes with persistent changes in expression; those were the genes which were continuously up- or down-regulated at 3 and 6 days after OPN knockdown. These genes included *RRM2* and *PVRL3*, which were down-regulated more than 1.5-fold. The third group of genes with compensated short-term changes included those genes which were modulated after 3 days of conditional OPN knockdown, but returned to the control levels of mRNA expression at 6 days after OPN knockdown. *IL11* qualified for this group, as it was slightly up-regulated after 3 days, but not modulated at 6 days after OPN inhibition (Figure 3). To confirm the above results, the modulations of *RRM2* and *PVRL3* were measured by qRT-PCR. Notably, these genes were persistently down-regulated by about twofold (see Figure S1). 

### 2.3. Breast Cancer Skeletal Metastasis Inhibition Following OPN Knockdown

**Figure 4:**
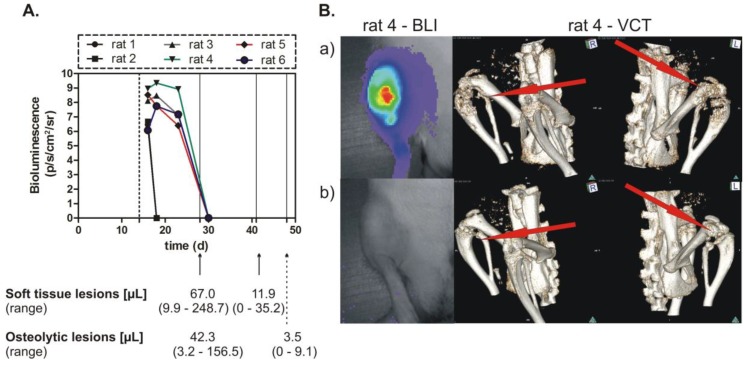
Inhibition of breast cancer skeletal metastasis upon conditional miRNA-mediated OPN knockdown. (**A**) Bioluminescence imaging (BLI) detection of tumor cells and measurement of the volume of soft tissue and osteolytic lesions. Cells of the clone O1 were inoculated into nude rats, which received doxycycline for 2 weeks (dashed line indicates the end of this administration) and were exposed to miRNA treatment thereafter. The time after tumor cell inoculation (days) is given on the *x*-axis; bioluminescence was measured in photons/second/cm^2^/steradian; the volume of soft tissue and osteolytic lesion is indicated at 28, 42 or 49 days after tumor cell inoculation or 14, 28, or 35 days of miRNA treatment. (**B**) BLI and volume computed tomography (VCT) images of tumors and skeletal metastasis. In the upper panel (**a**) are the BLI image (left) and VCT scans (middle and right), which show the status at 19 and 28 days after tumor cell inoculation, respectively; the lower panel (**b**) shows the BLI image (left) and VCT scans (middle and right), which indicate the status at 30 and 49 days after tumor cell inoculation, respectively.

In order to examine the effect of the conditional OPN knockdown on skeletal metastasis, the O1 cell clone was tested in a nude rat model of osteolytic metastasis. The reason for not using the O2 cell clone was that this clone did not show sufficient growth in vivo. The cells were inoculated into an experimental group of 6 rats, which were administered doxycycline per-orally, starting three days before tumor cell inoculation and ending 2 weeks thereafter. This period was needed in order to establish the breast cancer skeletal metastasis. Then, the tetracycline derivative was discontinued, thus starting the rats’ exposure to the miRNA targeting OPN. This treatment was continued for up to 5 weeks and the tumor sizes were followed by bioluminescence imaging (BLI). The soft tissue and osteolytic lesions which were established during the doxycycline intake period and the changes caused by the specific miRNA targeting OPN were assessed by MRI at 2 and 4 weeks and by volume computed tomography (VCT) at 2 and 5 weeks after the start of miRNA treatment. As indicated in Figure 4A, the BLI revealed complete remissions of the established tumors following different periods of time. The first and second rats had the same tumor size, detected for a short period of time. The soft tissue lesions were found decreased by 6 times after 2 weeks, and the osteolytic lesions by 12 times after 3 weeks. In addition, complete remissions of these lesions were detected. The bioluminescence images and VCT pictures revealed a strong anti-metastatic effect of OPN knockdown (Figure 4B).

### 2.4. OPN Present in the Exosomes

**Figure 5:**
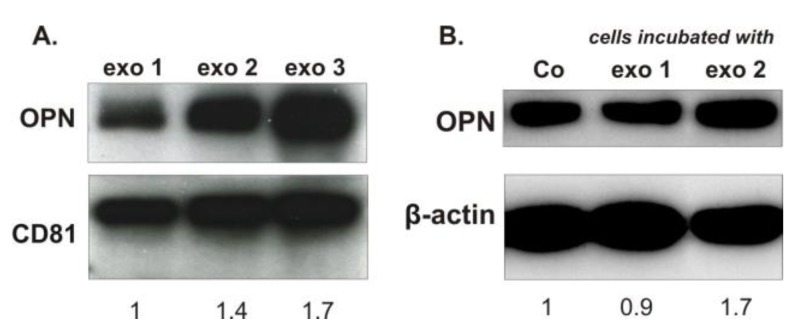
OPN protein levels in exosomes and in MDA-MB-231 cells incubated with these exosomes. (**A**) OPN was more abundant in exosomes isolated from the plasma of rats with breast cancer skeletal metastasis (exo 2 and exo 3) than in exosomes isolated from the plasma of healthy rats (exo 1). (**B**) OPN protein levels were increased in breast cancer cells incubated for 48 h with exosomes isolated from the plasma of rats with breast cancer skeletal metastasis (exo 2). “Co” indicates sample from control MDA-MB-231 cells, which were not incubated with any exosomes; CD81 and β-actin proteins were used as loading controls.

As exosomes are supposed to be one of the vehicles of proteins which could prepare the pre-metastatic niche or stimulate metastasis formation, we investigated the presence of OPN in two types of exosomes. First, isolated from the plasma of healthy rats (exo 1) and second, isolated from the plasma of rats with breast cancer skeletal metastasis (exo 2 and exo 3). In the latter exosomes, the respective levels of OPN were 1.4 and 1.7 times higher than in the control exosomes (Figure 5A). Moreover, 48 h incubation of MDA-MB-231 breast cancer cells with exo 2 led to 1.7 times increased OPN levels in these cells. Remarkably, exo 2 stimulated the migration of these cells by 27% after 72 h incubation in vitro (see Figure S2).

### 2.5. Effect of OPN Knockdown and Erufosine Treatment on Some PI3K/Akt Pathway Proteins in MDA-MB-231 Cells

**Figure 6:**
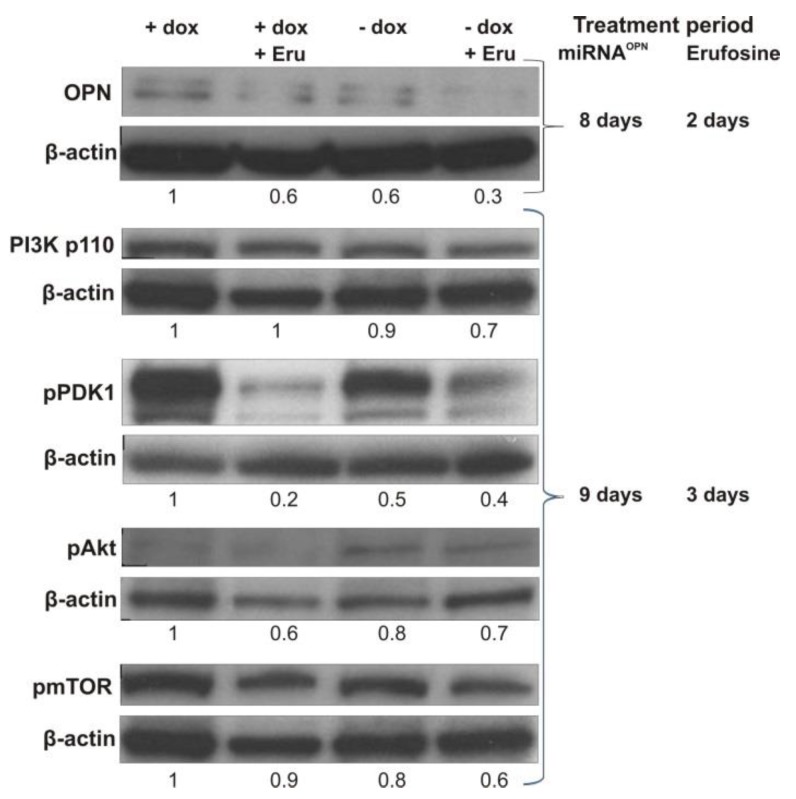
Effects of conditional OPN knockdown and exposure to erufosine alone or in combination on some PI3K/Akt pathway proteins in MDA-MB-231-derived breast cancer cells. Cells from the O2 cell clone were cultivated continuously in medium with doxycycline and were used as control (+dox) or were exposed to erufosine for 48 to 72 h (+dox + Eru). O2 cells were also cultivated in medium without doxycycline (i.e., they were treated with miRNA targeting OPN mRNA) for 8 or 9 days (−dox) or additionally they were exposed to erufosine for 48 to 72 h (−dox +Eru). miRNA^OPN^ indicates miRNA targeting OPN mRNA.

Considering that the PI3K/Akt pathway is very important in tumor progression, some proteins of this pathway were investigated upon conditional OPN knockdown, after erufosine treatment, or after their combination. Cells of the O2 cell clone showed 40% decreased OPN levels either upon conditional OPN knockdown for 8 days, or treatment by erufosine at IC50 (34 µM) for 48 h. However, the combination of both treatments led to an additive effect, as deduced from the reduction in OPN level by 70% (Figure 6). OPN knockdown together with erufosine treatment for 72 h caused a decrease of PI3K p110 by 30% and reduction of pmTOR by 40%. Erufosine alone produced the strongest reduction of pPDK1 (by 80%) and pAkt (by 40%) after 72 h of treatment. The combination of OPN knockdown and this drug also caused considerable decreases in the levels of these two proteins. 

## 3. Discussion

It has been reported that patients with advanced breast cancer and high expression of OPN have poor prognosis and shortened survival [9,10]. Additionally, elevated levels of OPN were exhibited in breast cancer bone metastasis [9], and it was hypothesized that this SIBLING protein could serve as a promising target for therapy [13]. As OPN is expressed by normal as well as tumor cells, there has been discussion about structural and/or functional differences between these OPN forms [9,12]. This could raise the question of whether OPN should be a target, regardless of its origin from normal and tumor cells, or whether its reduced production by tumor cells should be the only aim of treatment. To contribute to this question, we wanted to create a model in which OPN could be selectively knocked down in breast cancer cells, which cause osteolytic metastasis. 

Toward that aim, we generated two double transgenic MDA-MB-231 cell clones by RMCE, which allowed us to investigate the effect of a conditional, prolonged knockdown of OPN in vitro and in vivo. In the absence of doxycycline exposure to these cells, tTA activated the bidirectional Ptet-bi promoter, which then drove the simultaneous expression of a highly specific and efficient miRNA targeting OPN mRNA, as well as the reporter genes for firefly luciferase and red fluorescent protein mCherry. In the presence of doxycycline, the expression of these genes was switched off. The combination of the Tet-Off system with RNA interference allowed us to monitor the effect of the OPN knockdown starting at a definite time point and continuing this observation for a defined period of time. Moreover, the stimulation of mCherry expression upon removal of doxycycline from the cell medium indicated the concomitant expression of a specific miRNA, which could be followed easily by flow cytometry analysis. In addition, the expression of firefly luciferase in the absence of doxycycline allowed us to measure the tumor size by bioluminescence imaging in an animal model for breast cancer skeletal metastasis. Regarding the presence of eventual off-target effects, the specificity of the siRNA sequence used had been previously established. In these experiments, the most reliable siRNA had been selected and had caused less-pronounced but identical effects in vitro [13].

The cell clones generated by RMCE showed good regulation of mCherry expression by doxycycline. The conditional miRNA-mediated knockdown of OPN was related to a slight inhibition of proliferation, and after a longer time period to an intensive suppression of spontaneous migration and of colony formation. Most importantly, the decreased soft tissue and osteolytic lesions, as well as some complete remissions of such lesions, proved the efficiency of the conditional (doxycycline-dependent) prolonged OPN knockdown in vivo for time intervals of 4 to 5 weeks. 

The presented approach was used not only for observing pronounced effects of OPN knockdown in vitro and in vivo, but also for following the genes’ modulation in response to the reduced OPN levels. Toward that purpose, a gene expression profiling analysis was performed for the O2 cell clone after 3 or 6 days of OPN inhibition. Interestingly, the number of altered genes was relatively low, and the fold change of expression was not tremendously high. This could be explained by the relatively short time interval (6 days) of treatment and relatively long half-life of the OPN protein. When analyzing the efficacy of the miRNA against OPN, the expression of OPN at the protein level was not as decreased as the respective mRNA level. Therefore, the relatively moderate decrease (maximally 40%) could be reason for the low number of modulated genes and their slight changes in expression. This contrasts to some extent with our observations on the SIBLING protein bone sialoprotein II. Here, the respective miRNA caused a low effect at the mRNA level, but an at least 50% knockdown at the protein level and concomitantly a large number of genes were modulated at a distinct to intensive degree [15].

A progressive increase in expression was detected in *GOS2*, *c-FOS*, IGFBP1, and *SERPINB2*, and down-regulation of *RRM2* and *PVRL3* was observed. *IL11* was slightly up-regulated after only 3 days. Several studies have revealed the role of these genes. The G0/G1switch 2 gene (GOS2) encodes a mitochondrial protein that interacts with Bcl-2 and prevents its heterodimerization with Bax. Thus, it could induce apoptosis. Because GOS2 has pro-apoptotic activity and it is epigenetically repressed or down-regulated in human cancers, it could be described as a tumor-suppressor gene [16]. *c-FOS* is an immediate early gene that encodes a leucine zipper protein that can dimerize with proteins of the JUN family; together, they form the transcription factor complex AP1. In this regard, the FOS protein has been perceived as regulator of cell proliferation, differentiation, and transformation. However, in some cases the expression of FOS has been related to apoptosis [17], and its loss in human gastric and ovarian carcinomas was associated with disease progression. Although FOS was identified more than two decades ago, it still has its mysteries [18]. Depending on cell types and differentiation stages, c-FOS may act as a positive or negative regulator of cell growth [19]. The insulin-like growth factor binding protein 1 (IGFBP1) modulates the circulating levels of insulin-like growth factor I (IGF-I) by sequestering this protein [20,21], antagonizes its effect, and can induce apoptosis [21]. Moreover, IGFBP1 (at concentrations of 800 ng/mL or higher) can interact with integrin receptors to induce focal adhesion kinase (FAK) dephosphorylation, and the detachment and apoptosis of breast cancer cells [21]. SERPINB2 (serpin peptidase inhibitor) is one of the main inhibitors of urokinase plasminogen activator (uPA), which alters the proenzyme plasminogen into the serine protease plasmin that degrades many ECM components [22,23]. Therefore, uPA could be implicated in some pathophysiological processes, such as tumor progression and metastasis. High concentrations of SERPINB2 in a neoplastic tissue are associated with good prognosis in patients with breast, pancreatic, and ovarian cancers [22]. Ribonucleotide reductase (RR) is an enzyme that catalyzes the reduction of ribonucleoside diphosphates to deoxyribonucleoside diphosphates, and it has an important role for maintaining a pool of dNTPs for DNA synthesis and repair [24,25]. Therefore, RR has an important role in the regulation of cell proliferation [26]. RR has two subunits, M1 (RRM1) and M2 (RRM2), and the latter is overexpressed in human breast carcinoma tissue (DCIS) [25]. Furthermore, RRM2 can enhance angiogenesis by upregulating VEGF in oropharyngeal carcinoma cells, and thus anti-angiogenic and anti-tumor effects are anticipated following RRM2 inhibition [27]. The gene poliovirus receptor-related 3 (PVRL3) encodes the immunoglobulin-like cell adhesion protein nectin 3, which can form adherens junctions in epithelial cells in partnership with cadherin–catenin molecules [28]. Therefore, it may play a role in the adhesion process of tumor cells. Finally, interleukin 11 is a member of the gp130 family of cytokines. In general, it belongs to a group of genes which are essential for the metastatic colonization of a certain organ. More specifically, IL11 is an osteoclast-mobilizing factor which qualifies breast cancer cells to establish osteolytic metastasis in bone tissue [29].

Collectively, the expression profiling data following conditional OPN knockdown indicated an up-regulation of genes related to apoptosis (*GOS2*, *IGFBP1*), down-regulation of genes correlated with angiogenesis (*RRM2*) or tumor-cell adhesion (*PVRL3*), decrease of an osteolytic metastasis-associated gene (*IL11*), and up-regulation of a gene which counteracts uPA and is related to good prognosis in breast cancer patients (*SERPINB2*). Thus, the observed in vitro and in vivo data could be explained by the modulation of several genes after OPN knockdown. 

Exosomes and their formation by tumor cells have recently gained specific interest [14]. In this study, exosomes isolated from rats harboring MDA-MB-231-induced skeletal metastasis showed increased levels of OPN. Their quality as a horizontal carrier of signal molecules was confirmed by adding these exosomes to cells growing in vitro, which then produced significantly increased levels of OPN. These facts prompt us to speculate that an OPN knockdown in breast cancer cells would cause not only a decreased cellular production of OPN, but also a reduced level of OPN in the secreted exosomes. As it is known that tumor-derived exosomes can be taken up by cells from specific organs and prepare the pre-metastatic niche [14], we hypothesize that lowering the amount of OPN in these exosomes may decrease their ability to modulate the distant site of metastasis and decrease their capability for metastasis formation.

Erufosine is a third-generation alkylphosphocholine with remarkable antineoplastic properties [30,31,32,33]. Its efficacy is related to changes in the expression of certain components of vital signaling pathways (for review see [34]). Here, the combination of erufosine with conditional OPN knockdown was investigated. Interestingly, erufosine alone caused a reduction in OPN expression. The ultimate reason for this is unclear, but there is some interplay between the two actors. Erufosine decreases signaling via the RAS/RAF/MEK/ERK cascade [34]. On the other hand, OPN interacts with CD44 and integrins, as examples [12]. Therefore, reduced OPN levels would be responsible for decreased stimulation of the PI3K/Akt/mTOR and MEK/EKR/AP1 pathways. Co-treatment of miRNA leading to OPN knockdown with erufosine would therefore have a combination effect on decreasing signals for cell growth and survival, cell migration, and others. In fact, erufosine exposure of breast cancer cells undergoing miRNA-mediated inhibition of OPN expression caused an additive reduction effect on OPN levels in the investigated breast cancer cells. In addition, some proteins of the PI3K/Akt pathway, which are highly important in tumor progression, were decreased upon OPN knockdown, which further explains the observed anti-metastatic effects. Interestingly, the proteins of interest were more intensively decreased after applying the combination of conditional OPN inhibition and erufosine. The combined effect was additional in nature. 

In summary, conditional OPN knockdown in MDA-MB-231 breast cancer cell clones caused slight anti-proliferative, but more pronounced anti-migratory and anti-clonogenic effects in vitro, as well as partial and complete remissions of soft tissue and osteolytic lesions in a respective nude rat model. The modulations of some genes and proteins upon OPN knockdown, as well as the eventual decrease of the OPN amount in the tumor-derived exosomes, could explain the observed effects in vitro and in vivo. Finally, a combination of OPN inhibition and erufosine treatment could be an option for improving the anti-metastatic activity of OPN knockdown and could be used in the development of more efficient therapy of breast cancer skeletal metastasis.

## 4. Materials and Methods 

### 4.1. Cell Culture and the Generation of Double Transgenic Cell Clones

Parent breast cancer MDA-MB-231 cells and subclones were cultured in RPMI 1640 medium (Invitrogen, Karlsruhe, Germany) supplemented with 10% fetal calf serum (FCS), 100 ng/mL doxycycline (added to deactivate the tetracycline-controlled transactivator, tTA), 2 mM l-glutamine, 100 U/mL penicillin, and 100 µg/mL streptomycin (Invitrogen, Karlsruhe, Germany) in standard cell culture flasks (TPP, Trasadingen, Switzerland) in a humidified incubator at 37 °C and 5% CO_2_. The human breast adenocarcinoma cell line MDA-MB-231 was obtained from ATCC (Manassas, VA, USA).

Two breast cancer cell clones with conditional OPN knockdown (O1 and O2) were generated by recombinase-mediated cassette exchange (RMCE) from parent MDA-MB-231 cell clone by using plasmid constructs and by following a previously-described procedure [15].

### 4.2. Flow Cytometry Analysis

Each of the generated cell clones was maintained for 6 days in two small culture flasks (25 cm^2^) in medium with or without doxycycline. Then, the cells were harvested by trypsin/EDTA, resuspended by PBS (Dulbecco´s phosphate-buffered saline from Sigma-Aldrich, Steinheim, Germany), transferred into 5 mL tubes with cell-strainer cap (Becton Dickinson Labware, Heidelberg, Germany), and processed by an LSRII analyzer. These analyses were used to ensure the purity (>98%) of successfully transfected cell clones and to ensure the activation of the tet-off system in the absence of doxycycline [15].

### 4.3. RNA Isolation and Quantitative Polymerase Chain Reaction (qPCR)

For RNA isolation from O1 and O2 cell clones which had been cultivated in medium with or without doxycycline for 3 or 6 days, the RNeasy mini-kit (Qiagen, Hilden, Germany) was used. The collected cell pellets (2 × 10^5^–10 × 10^5^) were resuspended and lysed by appropriate volumes of RLT buffer. The next steps were performed according to the manufacturer’s recommendations and protocols. For determination of RNA amount and purity, samples were measured in a spectrophotometer using the absorption at 260 nm and the 260/280 ratio. For producing cDNA from the isolated RNA, a reaction mixture of 1000–1500 ng RNA, buffer RT (1×), dNTPs (0.5 mM), oligo-dT-primers (1 µM), RNAse inhibitor (20 units), and reverse transcriptase enzyme (8 units) was incubated in a total volume of 40 µL at 37 °C for 1 h. The cDNA synthesis was performed by using the Omniscript RT Kit according to the manufacturer’s protocol (Qiagen, Germany). The LightCycler 480 Real-Time PCR system and the human Universal Probe Library kit (Roche, Mannheim, Germany) were used following the manufacturer’s protocol. PCR Mix for 10 µL reaction was prepared by adding the necessary components: 2× (Buffer) LightCycler 480 Probes Master (5 µL), 10 µМ probe from the respective Universal Probe Library (UPL) probes (0.1 µL), 10 µM forward primer (0.2 µL), 10 µM reverse primer (0.2 µL), RNase/DNase-free water (2.5 µL). Eight microliters of PCR Mix was pipetted in triplicate into 384-well plates and 2 µL containing 50 ng cDNA per sample was added. Next, the prepared plate with the samples was centrifuged for 5 min at 4000 rpm and 4 °C and loaded into the Light Cycler 480 Instrument (Roche, Mannheim, Germany). The genes were amplified for 50 cycles, 30 s per cycle. The cDNA input was normalized to the expression of the house-keeping gene GAPDH.

### 4.4. Western Blot

Western blots were performed essentially as described in [32] with the following modifications: a protease inhibitor cocktail (Roche, 1:25) was used and no additional dithiothreitol was added to the sample mix including loading dye. Aliquots of cell lysates were further applied for Western blot analysis as published previously [15]. The following proteins were detected by specific antibodies: OPN (M66105M, Meridian Life Science, Memphis, TN, USA), PI3Kinase p110 β (#3011), pPDK1 (#3438), pAkt (#13038P), pmTOR (#5536) (Cell signaling technology, Frankfurt, Germany), and β-actin (sc1615, Santa Cruz, Heidelberg, Germany), which served as internal loading control. The respective horseradish-peroxidase-conjugated secondary antibodies were donkey anti-goat (sc2020), goat anti-mouse (sc2055) (Santa Cruz), and goat anti-rabbit (#7074, Cell signaling technology). 

### 4.5. Cell Proliferation, Migration, and Colony Formation Assays

Proliferation of tumor cells was determined by MTT assay. Here, 2 × 10^3^–3 × 10^3^ cells/well were seeded into 6-well plates and cultivated for 3 and 6 days in media with or without doxycycline. Then, 400 µL MTT solution (5 mg/mL Sigma, St. Louis, MO, USA dissolved in PBS) was added to each well and incubated for 3.5 h at 37 °C. The yellow MTT was reduced to purple formazan by dehydrogenases in the mitochondria of the living cells. Subsequently, the formazan crystals were solubilized with 0.04 M HCl-isopropanol. Optical density was measured at 540 nm wavelength (690 nm reference wavelength) using an ELISA plate reader (Anthos Mikrosysteme, Krefeld, Germany). 

The migration assay setting and procedure was as described previously [15]. Briefly, control and specific cell clones with the conditional expression of miRNA targeting OPN were seeded for 9 days in culture flasks with or without doxycycline-containing media. Then, the cells were harvested, re-suspended in media without FCS, and seeded in equal amount (1 × 10^5^) into inserts with 8 µm pore size membranes (Millicell, Millipore, Switzerland) (upper compartment). The lower compartment contained media with 10% FCS and the cells were subsequently allowed to migrate into it for 2 or 3 days. The migrated cells were quantified using Cell Titer Blue Reagent (Promega, Mannheim, Germany), according to the manufacturer’s protocol.

The colony formation assay was performed as published previously [15]. Here, the control and specific cell clones (O1 and O2) were seeded for 6 and 9 days in media with or without doxycycline. Briefly, the cells were harvested, equal amounts of them (1.25 × 10^3^) were added to semi-solid mixtures (0.8% methylcellulose, +/− doxycycline, 40% FCS) and kept under standard cell culture conditions (37 °C, 5% CO_2_ in humidified air). The colonies were counted under an inverted microscope after 7 days.

### 4.6. Animals

Male nude rats (RNU strain, Charles River, Germany) were obtained at an age of 4 to 6 weeks and kept under specific-pathogen-free (SPF) conditions in Macrolon-IV-cages of a ventilated rack (Ventirack, UNO Roestvaststaal B.V., Zevenaar, The Netherlands) providing a 50-fold exchange of filtered air per hour as well as positive air pressure inside the cages. They were maintained under controlled conditions—constant room temperature (22 ± 1 °C), air humidity (50% ± 10%) and dark–light rhythm (12 h–12 h). The animals had free access to autoclaved water and standard laboratory diet. Experiments were started after a 1-week adaptation period. All animal experiments were approved by the responsible governmental animal ethics committee (Regierungspräsidium Karlsruhe, Germany [35-9185.81-G70/12]). 

### 4.7. Ethics Statement

The investigation involving animals was conducted in accordance with the commonly accepted “3Rs” (i.e., replacement of animals by alternatives wherever possible, reduction in the number of animals used, and refinement of the experimental conditions and procedures to minimize the harm to animals) and according to national and international guidelines, and has been approved by the authors’ institutional review board.

### 4.8. Tumor Cell Inoculation

The experimental rats received doxycycline via their drinking water (2 µg/mL) three days before tumor cell inoculation. Sodium cyclamate (240 mg/L) and saccharin-sodium (24 mg/L) were added to the drinking water to hide the bitter taste of doxycycline. The preparation of the cell suspensions and the subsequent tumor cell inoculation into the saphenous artery of the rats were done as described previously [15,35].

### 4.9. Setup of Animal Experiments and Tumor Size Determination

The cell clone with conditional expression of miRNA targeting OPN (O1 cell clone) was tested in a group of six experimental rats receiving doxycycline (2 µg/mL) for 2 weeks who were then exposed to a specific miRNA against OPN for up to 5 weeks by discontinuation of doxycycline. Bioluminescence imaging (BLI), magnetic resonance imaging (MRI), and volume computed tomography (VCT) were used to follow the tumor size in the experimental animals [35].

### 4.10. Microarray Analysis

Microarray analysis was performed as described [36]. Microarray scanning was done using an iScan array scanner. As a test for significance, the Student’s *t*-test was used on the bead expression values of the two groups of interest. The average expression value was calculated as the mean of the measured expressions of beads together with the standard deviation of the beads. Modulations in gene expression were considered significant if the *p*-value corrected by the Benjamini–Hochberg procedure was less than 0.01. This was observed for all modulations exceeding a 1.5-fold change.

Entry name/accession number: The dataset was submitted to the Gene Expression Omnibus (GEO) database. The corresponding number is GSE133608.

### 4.11. Exosomes Isolation and Lysis

The exosomes of the plasma of healthy rats and of rats harboring breast cancer skeletal metastasis were isolated by ExoEasy Maxi Kit (Qiagen) according to the manufacturer’s recommendations and protocol. Then, an aliquot of exosomes (60 µL) was lysed by 5× RIPA buffer containing protease inhibitors (15 µL) on a rotor at 4 °C for 30 min. Afterwards, the lysates were centrifuged at 14,000 rpm for 20 min at 4 °C. The proteins in the supernatant were further analyzed by Western blot.

### 4.12. Treatment of O2 Cell Clone by Erufosine

Cells from the O2 cell clone were seeded in media with or without doxycycline for 8 or 9 days. On the sixth day, they were treated by erufosine at IC50 (34 µM) for 48 or 72 h. After these periods of time, the cells were collected for further Western blot analysis. 

### 4.13. Statistics

The data of multiple measurements from in vitro functional tests were presented as means with corresponding standard deviations. The independent manifestation of investigated parameters (in vitro functional properties) was examined by two-way analysis of variance (ANOVA) test and Bonferroni post-hoc test. A *p*-level less than 0.05 was considered significant.

## Figures and Tables

**Table 1:** Overview of gene expression modulation following conditional osteopontin knockdown.

Changes in Gene Expression	Significant Modification ^a^ Day 3 Day 6		Number of Genes	Percentage of Genes with More Than 1.5-Fold Change in Expression
Compensated short-term	↓ ^b^	- ^d^	3	7.1%
	↑ ^c^	-	9	21.4%
Persistent	↓	↓	3	7.1%
	↑	↑	1	2.4%
Long-term	-	↓	12	28.6%
	-	↑	14	33.4%

^a^ Fold change in expression >1.5 and <−1.5fold; ^b^ Significant down-regulation; ^c^ Significant up-regulation; ^d^ Less than 1.5-fold modulation was disregarded.

## Supplementary Materials

Supplementary materials can be found at https://www.mdpi.com/1422-0067/20/19/4918/s1.
