# Supplementary material for: Conditional Knockdown of Osteopontin Inhibits Breast Cancer Skeletal Metastasis
Source: Int J Mol Sci. 2019 Oct 4;20(19):4918. doi: 10.3390/ijms20194918 (PMC6801824; doi:10.3390/ijms20194918)
Supplement: Supplementary file 1 [file ijms-20-04918-s001.pdf]

Supplementary Figures

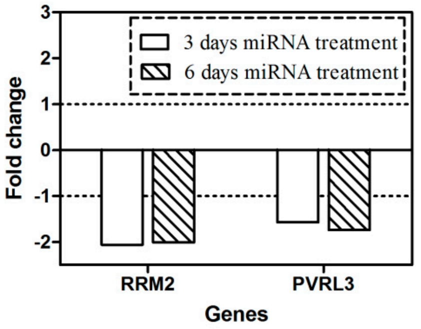

Supplementary Figure S1. Gene expression analysis by quantitative real-time PCR. Fold changes of selected genes' expression in response to targeting OPN by miRNA for 3 or 6 days. RRM2 and PVRL3 showed persistent downregulation. Dashed lines indicate the no-effect level regarding gene expression modulation.

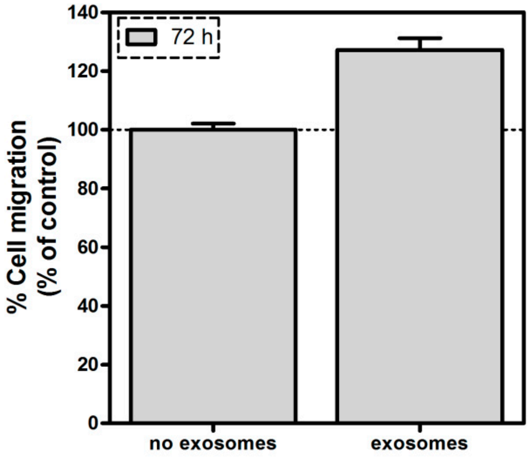

Supplementary Figure S2. Migration of MDA-MB-231 cells with or without the addition of exosomes. Exosomes isolated from plasma of rats with breast cancer skeletal metastasis stimulated the migration of MDA-MB-231 cells after 72 h incubation.
